# Providing COVID-19 vaccination to refugees and displaced people: lessons from the vaccine roll-out for the Rohingya refugees in Cox's Bazaar, Bangladesh

**DOI:** 10.1016/j.lansea.2022.100120

**Published:** 2022-11-23

**Authors:** Ali M. Alam

**Affiliations:** Infection Neuroscience Lab, Department of Clinical Infection, Microbiology, & Immunology, Institute of Infection Veterinary and Ecological Sciences, University of Liverpool, Liverpool, UK

In the global vaccination efforts against COVID-19, there is a general concern of refugees being left behind.^1^ A vaccination campaign was held in the world's largest refugee camp in Cox's Bazaar, Bangladesh where almost 900,000 Rohingya (Forcibly Displaced Myanmar Nationals or FDMN) people reside. Within four months, 86% of FDMN above 55 years of age had been vaccinated, which is a remarkable feat.^2^ What lessons can we learn from this campaign that has seen a high uptake of vaccination in a marginalised population with a history of limited health seeking behaviour?

The WHO played a major role in the campaign by citing the importance of refugee populations in receiving vaccinations and lobbied the Bangladeshi government to revise the vaccination plan to include the FDMN as an objective group.^3^ Although around 130,000 vaccines were specifically earmarked for distribution among the FDMN in March 2021, it was not until August that the first vaccine was administered.^4^ The failure to hit this target could be majorly attributed to lack of supply. Bangladesh was due to receive 12 million doses in early 2021 through the COVAX initiative, but by May 2021 the country had not received a single dose.^5^ These shortages led to fears that Bangladesh would prioritise the limited number of available vaccines and the promises to vaccinate refugees would not be materialised. However, the WHO advocated the countries with surplus doses to donate to COVAX and for manufacturers to boost supplies to the COVAX manufacturing facilities.^4^ This led to an upscaling of supply and Bangladesh has now seen over 190 million doses delivered through COVAX and bilateral donations. Increasing the supply to Bangladesh has been vital in vaccination of FDMN and the global community must continue to support schemes like COVAX in delivering doses to low-income and middle-income countries, where 85% of refugees are hosted, so that they need not prioritise their vaccines to the detriment of displaced populations.

Over 150 states have adopted COVID-19 vaccine strategies to include refugees—but inclusion in policy does not always translate into equitable access.^6^ Marginalised groups are more likely to experience barriers in accessing healthcare, which in turn leads to vaccine hesitancy. Prior to the pandemic, studies reflected vaccine hesitancy among FDMN, with resistance from religious leaders and gender barriers being the main contributing factors.^7^ These issues, paired with a general mistrust of local health systems, must be overcome to ensure adequate vaccine uptake in marginalised communities. To resolve this, governments need to invest in qualitative research aiming to understand the needs of the culturally and linguistically unique displaced communities.^8^ Engagement with community leaders ahead of vaccination campaigns has been vital in Cox's Bazaar, as well as educational programmes combatting identified misinformation among the FDMN.^9^ For example, information gathered from initial community-level consultations was used develop to outreach programmes sensitive to the thoughts and feelings of the FDMN.^9^ Vaccination centres were supported by Rohingya volunteers, who had a key role in communicating health messages, liaising with community leaders, and accompanying older people to vaccination centres.^8^^,^^10^ The inclusion of the Rohingya people themselves in vaccine outreach programmes helped to foreground cultural consideration whilst tackling vaccine hesitancy.

Marginalised groups remain vulnerable to the threat of COVID-19 and the world-wide community must ensure displaced populations will not be left behind in the global vaccination drive. The vaccine programme for the FDMN in Cox's Bazaar illustrates how equitable vaccine access and culturally sensitive vaccine outreach programmes contributes to overall success of global vaccination efforts.

## Contributors

AMA conceptualised and wrote the paper.

## Declaration of interests

None.
